# The influence of acculturation on the risk of preterm birth and low birthweight in migrant women residing in Western Australia

**DOI:** 10.1371/journal.pone.0285568

**Published:** 2023-05-10

**Authors:** Maryam Mozooni, Gavin Pereira, David Brian Preen, Craig Edward Pennell

**Affiliations:** 1 Discipline of Obstetrics and Gynaecology, Medical School, The University of Western Australia, Perth, WA, Australia; 2 School of Population and Global Health, The University of Western Australia, Perth, WA, Australia; 3 School of Medicine, The University of Notre Dame, Fremantle, Western Australia, Australia; 4 Curtin School of Population Health, Curtin University, Perth, Western Australia, Australia; 5 enAble Institute, Curtin University, Perth, Western Australia, Australia; 6 Centre for Fertility and Health (CeFH), Norwegian Institute of Public Health, Oslo, Norway; 7 School of Medicine and Public Health, The University of Newcastle, Callaghan, NSW, Australia; PLOS (Public Library of Science), UNITED KINGDOM

## Abstract

**Background:**

The risk of preterm birth (PTB) and low birthweight (LBW) may change over time the longer that immigrants reside in their adopted countries. We aimed to study the influence of acculturation on the risk of these outcomes in Australia.

**Methods:**

A retrospective cohort study using linked health data for all non-Indigenous births from 2005–2013 in Western Australia was undertaken. Acculturation was assessed through age on arrival, length of residence, interpreter use and having an Australian-born partner. Adjusted odds ratios (aOR) for term-LBW and PTB (all, spontaneous, medically-indicated) were calculated using multivariable logistic regression in migrants from six ethnicities (white, Asian, Indian, African, Māori, and ‘other’) for different levels of acculturation, compared to the Australian-born population as the reference.

**Results:**

The least acculturated migrant women, those from non-white non-Māori ethnic backgrounds who immigrated at age ≥18 years, had an overseas-born partner, lived in Australia for < 5 years and used a paid interpreter, had 58% (aOR 1.58, 95% CI 1.15–2.18) higher the risk of term-LBW and 40% (aOR 0.60, 95% CI 0.45–0.80) lower risk of spontaneous PTB compared to the Australian-born women. The most acculturated migrant women, those from non-white non-Māori ethnic backgrounds who immigrated at age <18 years, had an Australian-born partner, lived in Australia for > 10 years and did not use an interpreter, had similar risk of term-LBW but 43% (aOR 1.43, 95% CI 1.14–1.78) higher risk of spontaneous PTB than the Australian-born women.

**Conclusion:**

Acculturation is an important factor to consider when providing antenatal care to prevent PTB and LBW in migrants. Acculturation may reduce the risk of term-LBW but, conversely, may increase the risk of spontaneous PTB in migrant women residing in Western Australia. However, the effect may vary by ethnicity and warrants further investigation to fully understand the processes involved.

## Introduction

Preterm birth (PTB), birth before 37 completed weeks of pregnancy [1], and low birthweight (LBW), birthweight less than 2500 grams regardless of the gestational age of the newborn [2], are more common among some migrant groups than the non-migrant population in high-income countries (HICs), such as Australia, the United Kingdom (UK) and the United States (US) [3, 4]. These adverse pregnancy outcomes are associated with considerable financial costs imposed on families and healthcare systems [5, 6] and result in higher rates of mortality and morbidity in childhood and throughout life [7].

Culture can influence a range of behavioural factors including diet [8], smoking in pregnancy [9, 10], and physical activity [11] that are associated with birthweight and other adverse pregnancy outcomes [12–14]. Further, a person’s culture can be influenced by the social environment in which they live and can change due to encounters with other cultures after immigration; this is known as ‘acculturation’ [15]. Mounting evidence from the US and Europe suggests that acculturation may influence the risk of adverse pregnancy outcomes [9, 16–21]. We previously investigated the influence of acculturation on the risk of stillbirth [22]; however, the effect of acculturation on the risk of LBW and PTB in migrant populations is not well understood in Australia.

The objective of this study was to investigate the influence of age on arrival, length of residence, using an interpreter and having an Australian-born partner, as proxies for acculturation, on disparities observed in the risk of PTB and term-LBW between Australian- and overseas-born populations from diverse ethnic backgrounds.

## Materials and methods

### Study population and data sources

Using routinely collected administrative health and registry data from the Department of Health Western Australia (WA) linked through the WA Data Linkage System (WADLS) [23–25] we undertook a retrospective cohort study. De-identified administrative data for the entire non-Indigenous population of singleton live births in WA from 1 January 2005 to 31 December 2013 were used.

The WADLS links data from a variety of health and other administrative datasets using best-practice probabilistic matching based on full name and address, phonetic compression algorithms and other identifiers [23–25]. Further, it has designed and uses numerous automated and manual sub-processes specifically to reduce the likelihood of linkage error—the details of which are reported in linkage quality statements published elsewhere [26, 27].

Primarily, data were sourced from the Midwives Notification System (MNS). The MNS is a statutory database, including maternal, pregnancy, birth and newborn information collected by attending midwives for all births of at least 20 weeks gestation or at least 400 grams in weight (if gestational age was unknown) in WA. We supplemented the data from the MNS with additional variables linked from the WA Birth Register, including maternal, paternal and infant demographic characteristics for all registered births in WA, and also from the Hospital Morbidity Data Collection (HMDC) which contains information related to inpatient separations from all WA private and public hospitals [28]. We used the Family Connections Linkage Facility [29] of the WADLS to genealogically link migrant women with their child outcomes.

### Variables

PTB was defined as birth before 37 completed weeks of gestation [1], and LBW as birthweight less than 2500 grams at the time of birth [2].

Birth status (live birth, stillbirth), plurality (number of babies in the index birth), infant weight (grams) and estimated gestational age at time of birth (completed weeks), were all extracted from MNS data and used to identify live singleton births and LBW and PTB outcomes. Term-LBW cases were identified by further consideration of the estimated gestational age at time of birth (in completed weeks). Onset of labour with values spontaneous/induced/No-labour was used to separate PTB cases as spontaneous or medically-indicated PTB.

Migrant status (Australian- or overseas-born), using mother’s place/country of birth (99.0% from Birth Registrations, 1.0% from HMDC) regardless of type of visa or reason for immigration, and self-reported ethnic origin (100% from MNS) were used to categorise the study population into Australian-born and migrants from white, Asian, Indian, African, Māori, or other ethnic backgrounds [30].

Year of birth (of the baby), maternal age (<20, 20–24, 25–29, 30–34, 35–39, 40–44, >44 years), maternal height (≤150 cm, 150–165 cm, >165 cm), marital status (never married, divorced/separated, married/de facto, other), parity (nulliparous, primiparous, multiparous), smoking in pregnancy (Yes/No), history of pre-existing medical conditions such as hypertension and/or diabetes (Yes/No), gestational diabetes (Yes/No), preeclampsia (Yes/No), fertility treatment (Yes/No), infant sex, and socioeconomic status using the area-based Index of Relative Socioeconomic Disadvantage (IRSD) which summarises a range of information about the economic and social conditions (such as qualification, income and occupation) of people and households within a geographical area [22], were all extracted from the MNS.

Length of residence in WA and age on arrival were calculated using mother’s year of arrival (4.5% missing values) and baby’s year of birth. Status of having an Australian-born partner (Yes/No) was derived from father’s place of birth from Birth Registration data (with 3.3% missing values). Paid interpreter utilisation (Yes/No) and private health insurance (Yes/No) were retrieved from HMDC records that were available for 99.0% of the whole population of births in WA. Level of acculturation was first examined by creating a new variable that had five following indicators; Australian-born women, Migrant women with overseas-born partner and interpreter, Migrant women with overseas-born partner but no interpreter, Migrant women with Australian-born partner, Others. Then, the “least acculturated” migrant women were defined as non-white-non-Māori women with a length of residence of <5 years who had an overseas-born partner, immigrated as an adult and utilised an interpreter when navigating the healthcare system; and non-white-non-Māori women who lived in Australia >10 years, immigrated as a child, had an Australian-born partner and did not have an interpreter in hospital were considered “the most acculturated” migrant women. This was examined in a separate analysis by creating a new variable, acculturated, with the following indicators: Australian-born, the least acculturated migrants, the most acculturated migrants, and other migrants.

### Statistical methods

The demographic and obstetric characteristics of the study populations were summarised and reported by percentage. Logistic regression was used to calculate the odds ratios (OR) and 95% confidence intervals (CI) for associations between level of acculturation and term-LBW and PTB (spontaneous and medically-indicated), using separate models for each outcome. Statistical significance was set at P<0.05 and analyses were adjusted for year of birth, maternal age group, marital status, maternal height, parity, socioeconomic disadvantage, infertility treatment, pre-existing medical condition, private health insurance, infant sex, and smoking in pregnancy [31–33] that were significant in univariate logistic regressions. In multivariable logistic regression, significant co-variables and confounders were selected a priori. The choice of the variables was also confirmed by using Directed Acyclic Graphs (DAGs) the details of which can be found in S1 File. When specific ethnic groups were investigated in sub-group analyses, due to the small numbers, the analyses were only adjusted for maternal age-groups and smoking in pregnancy. The proportion of missing data was low (<10%) except for maternal height that was on average 12%. Those with a missing value were categorized as a separate subgroup to retain all the cases in the analysis.

All the analyses were performed using Stata (version 13·1; StataCorp LP, College Station, Texas).

Ethics approval for this study was granted by the Human Research Ethics Committee of the WA Department of Health (reference, 2015/23). Written consent from participants was not required to conduct the study due to the use of non-identifiable routinely collected linked administrative health data for the whole population.

## Results

### Descriptive analysis

From the 252 256 live singleton births studied, 33.9% occurred among the migrant mothers and 66.1% among the women born in Australia (Table 1). Migrant women were slightly older than the Australian-born group (mean age in years: 30.9 vs 29.5) and had a higher proportion of married women than the Australian-born population (91.7% vs 88.0%). Migrant women were also less likely to smoke in pregnancy (7.1% vs 14.0%), fall in the most socioeconomically disadvantaged category (13.6% vs 23.5%) or have pre-existing medical conditions (31.2% vs 35.0%), but were more likely to experience gestational diabetes than their Australian-born counterparts (8.1% vs 4.4%). Among migrant women, 30% had an Australian-born partner, 55.0% arrived in Australia as an adult, and 40.3% gave birth in their first five years of residence, including 12.1% who gave birth in the first two years after immigrating to Australia.

**Table 1:** Characteristics of the study population.

Characteristics	Australian-born women	Migrant women							All women
	All	White	Asian	Indian	African	Māori	Other	All	
**Live & singleton births**	166 775	46 878	17 734	5352	3985	2867	8665	85 481	252 256
	(66.1%)	(18.6%)	(7.0%)	(2.1%)	(1.6%)	(1.1%)	(3.4%)	(33.9%)	(100%)
**PTB**	10 517	2699	1177	387	227	169	564	5223	15 741
	(6.3%)	(5.8%)	(6.6%)	(7.2%)	(5.7%)	(5.9%)	(6.5%)	(6.1%)	(6.2%)
**LBW**	6539	1830	962	429	221	136	477	4055	10 923
	(4.1%)	(3.9%)	(5.4%)	(8.0%)	(5.6%)	(4.7%)	(5.5%)	(4.7%)	(4.3%)
*Maternal height*									
**≤150cm**	1442	539	1524	327	64	16	384	2854	4296
	(0.9%)	(1.2%)	(8.6%)	(6.1%)	(1.6%)	(0.6%)	(4.4%)	(3.3%)	(1.7%)
**150-165cm**	72 734	21 706	12 605	3892	2037	1236	5455	46 931	119 665
	(43.6%)	(46.3%)	(71.1%)	(72.7%)	(51.1%)	(43.1%)	(64.0%)	(54.9%)	(47.4%)
**>165cm**	72 060	18 607	1885	681	1514	1236	2093	26 016	98 076
	(43.2%)	(39.7%)	(10.6%)	(12.7%)	(38.0%)	(43.1%)	(24.2%)	(30.4)	(38.9%)
**Missing**	20 539	6026	1720	452	370	379	733	9680	30 219
	(12.3%)	(12.9%)	(9.7%)	(8.5%)	(9.3%)	(13.2%)	(8.5%)	(11.3%)	(12.0%)
*Marital status*									
**Never married**	17 381	2933	689	99	515	554	586	5376	22 759
	(10.4%)	(6.2%)	(3.9%)	(1.8%)	(13.1%)	(19.4%)	(6.8%)	(6.3%)	(9.0%)
**Divorced/separated**	1492	347	154	17	102	31	122	773	2266
	(0.9%)	(0.7%)	(0.9%)	(0.3%)	(2.6%)	(1.1%)	(1.5%)	(0.9%)	(0.9%)
**Married/de facto**	146 765	43 144	16 647	5176	3317	2214	7876	78 374	22 5164
	(88.0%)	(92.1%)	(93.4%)	(96.7%)	(83.2%)	(77.2%)	(90.1%)	(91.7%)	(89.3%)
**Other**	1137	454	244	60	51	68	81	958	2095
	(0.7%)	(1.0%)	(1.4%)	(1.1%)	(1.3%)	(2.4%)	(0.9%)	(1.1%)	(0.8%)
*Parity*									
**Nulliparous**	70 996	20 466	8519	3114	1175	932	3360	37 566	108 571
	(42.6%)	(43.7%)	(48.0%)	(58.2%)	(29.5%)	(32.5%)	(38.8%)	(44.0%)	(43.0%)
**Primiparous**	58 475	16 658	6326	1768	1069	770	2619	29 210	87 691
	(35.1%)	(35.5%)	(35.7%)	(33.0%)	(26.8%)	(26.9%)	(29.8%)	(34.2%)	(34.8%)
**Multiparous**	37 304	9754	2889	470	1741	1165	2686	18 705	56 015
	(22.4%)	(20.8%)	(16.3%)	(8.8%)	(43.7%)	(40.6%)	(31.0%)	(21.9%)	(22.2%)
*Maternal age*									
**Mean (SD)**	29.5	31.5	31.2	29.5	28.8	26.8	29.9	30.9	30.0
	(5.6)	(5.3)	(4.9)	(4.4)	(5.7)	(6.0)	(5.6)	(5.4)	(5.6)
**<20**	7312	703	131	17	188	297	193	1529	8842
	(4.4%)	(1.5%)	(0.7%)	(0.3%)	(4.7%)	(10.4%)	(2.2%)	(1.8%)	(3.5%)
**20–24**	26 849	4254	1383	629	793	862	1439	9365	36 216
	(16.1%)	(9.1%)	(7.8%)	(11.8%)	(20.0%)	(30.1%)	(16.6%)	(11.0%)	(14.4%)
**25–29**	47 602	11 150	5099	2228	1209	766	2546	22 998	70 607
	(28.5%)	(23.8%)	(28.8%)	(41.6%)	(30.3%)	(26.7%)	(29.4%)	(26.9%)	(28.0%)
**30–34**	52 740	16 874	6751	1816	1116	591	2590	29 738	82 492
	(31.6%)	(36.0%)	(38.1%)	(33.4%)	(28.0%)	(20.6%)	(29.9%)	(34.8%)	(32.7%)
**35–39**	27 172	11 184	3587	557	556	277	1522	17 683	44 859
	(16.3%)	(23.9%)	(20.2%)	(10.4%)	(14.0%)	(9.6%)	(17.6%)	(20.7%)	(17.8%)
**40–44**	4929	2590	754	101	107	74	360	3986	8915
	(2.3%)	(5.5%)	(4.3%)	(1.9%)	(2.7%)	(2.6%)	(4.2%)	(4.7%)	(3.5%)
**>44**	171	123	29	<10	11	0	15	182	353
	(0.1%)	(0.3%)	(0.2%)	(0.1%)	(0.3%)	(0.0%)	(0.2%)	(0.2%)	(0.1%)
**Australian-born Partner**	115 463	19 130	3454	257	168	627	1482	25 118	140 581
	(71.8%)	(41.7%)	(19.7%)	(4.8%)	(4.4%)	(23.5%)	(17.6%)	(30.0%)	(57.5%)
*Age on arrival*									
**<18 years old**	NA	22 516	6021	675	1087	1499	2850	34 648	NA
		(48.0)	(34.0%)	(12.6%)	(27.3%)	(52.3%)	(32.9%)	(40.5%)	
**18 or older**	NA	22 744	10 793	4420	2569	1226	5277	47 029	NA
		(48.5%)	(60.9%)	(82.6%)	(64.5%)	(42.8%)	(60.9)	(55.0%)	
*Length of residence*									
**<2years**	NA	3961	2316	1282	712	519	1543	10 333	NA
		(8.5%)	(13.1%)	(24.0%)	(17.9%)	(18.1%)	(17.8%)	(12.1%)	
**2–5 years**	NA	11 162	5302	2570	1556	839	2710	24 139	NA
		(23.8%)	(29.9%)	(48.0%)	(39.1%)	(29.3%)	(31.3%)	(28.2%)	
**>5 years**	NA	30 123	9192	1243	1383	1367	3872	47 180	NA
		(64.3%)	(51.8%)	(23.2%)	(34.7%)	(47.7%)	(44.7%)	(55.2%)	
**Unknown**	NA	1632	924	257	334	142	540	3829	NA
		(3.5%)	(5.2%)	(4.8%)	(8.4%)	(5.0%)	(6.2%)	(4.5%)	
**Any medical condition**	58 385	14 255	4744	2038	1489	974	3163	26 663	85 054
	(35.0%)	(30.4%)	(26.8%)	(38.1%)	(37.4%)	(34.0%)	(36.5%)	(31.2%)	(33.7%)
**Infertility treatment**	5108	1608	395	120	33	15	153	2324	7432
	(3.1%)	(3.4%)	(2.2%)	(2.2%)	(0.8%)	(0.5%)	(1.8%)	(2.7%)	(3.0%)
**Smoked**	23 310	4010	313	40	70	1125	465	6023	29 336
	(14.0%)	(8.6%)	(1.8%)	(0.8%)	(1.8%)	(39.2%)	(5.4%)	(7.1%)	(11.6%)
Most disadvantaged*	39 242	6326	2140	642	491	777	1237	11 613	50 858
	(23·5%)	(13·5%)	(12·1%)	(12·0%)	(12·3%)	(27·1%)	(14·3%)	(13.6%)	(20·2%)
**Gestational diabetes**	7316	2612	2243	834	297	115	822	6931	14 239
	(4.4%)	(5.6%)	(12.7%)	(15.6%)	(7.5%)	(4.0%)	(9.5%)	(8.1%)	(5.6%)
**Preeclampsia**	4650	1053	298	119	116	73	198	1857	6507
	(2.8%)	(2.3%)	(1.7%)	(2.2%)	(2.9%)	(2.6%)	(2.3%)	(2.2%)	(2.6%)
*Infant sex*									
**Male**	85 285	23 882	9207	2729	2055	1450	4435	43 758	129 057
	(51.1%)	(51.0%)	(51.9%)	(51.0%)	(51.6%)	(50.6%)	(51.2%)	(51.2%)	(51.2%)

*The bottom 20% of IRSD scores indicate the area with greatest disadvantage in terms of people with low income, no qualifications, and low skill occupations

### Acculturative factors

Lower length of residence in Australia, immigrating as an adult and having an Australian-born partner were slightly associated with lower odds of PTB (Table 2) while using the interpreter service was strongly associated with lower odds of this outcome (aOR 0.58, 95% CI 0.49–0.69). Using an interpreter had the most protective effect among women from African background (OR 0.31, 95% CI 0.17–0.52).Each year increase in the length of residence was associated with 1% decrease in the odds of Term LBW (OR 0.993, 95% CI 0.991–0.994) and 5% increase in the odds of PTB (OR 0.05, 95% CI 1.004–1.006).

**Table 2:** Comparison of Term-LBW and PTB in migrants, stratified by acculturative factors, with Australian-born women.

Acculturative factor	*N*	Term-LBW		All PTB	
		OR (95% CI)	aOR (95% CI)**	OR (95% CI)	aOR (95% CI)**
**Australian-born (Reference)**		1.00	1.00	1.00	1.00
**Overseas-born**		1.35*(1.27–1.45)	1.34*(1.24–1.44)	0.98 (0.95–1.01)	0.94*(0.90–0.97)
*Interpreter use*					
**Non-white non-Māori migrant with interpreter**	3493 (9.8%)	1.58*(1.24–2.01)	1.60*(1.24–2.06)	0.64*(0.55–0.76)	0.58*(0.49–0.69)
**Non-white non-Māori migrant without interpreter**	32 066 (90.2%)	1.85*(1.70–2.02)	1.84*(1.67–2.03)	1.09*(1.04–1.14)	1.02 (0.97–1.08)
*Length of residence*					
**<5 years**	29 392 (34.4%)	1.51*(1.37–1.66)	1.47*(1.33–1.63)	0.93*(0.88–0.98)	0.91*(0.86–0.96)
**5–10 years**	17 844 (20.9%)	1.22*(1.07–1.39)	1.25*(1.09–1.42)	0.95 (0.89–1.01)	0.92*(0.86–0.99)
**>10 years**	34 416 (40.3%)	1.22*(1.11–1.35)	1.22*(1.10–1.35)	1.00 (0.95–1.05)	0.96 (0.91–1.01)
*Age on arrival*					
**≥18 years old**	53 555 (65.6%)	1.38*(1.27–1.49)	1.42*(1.31–1.55)	0.94*(0.90–0.98)	0.92* (0.88–0.96)
**<18 years old**	28 074 (34.4%)	1.25*(1.12–1.39)	1.21*(1.09–1.35)	1.00(0.95–1.05)	0.97 (0.92–1.02)
**Australian-born partner**					
**Migrant with overseas-born partner**	60 363 (70.6%)	1.49*(1.39–1.61)	1.49*(1.38–1.62)	0.96*(0.92–1.00)	0.92*(0.89–0.96)
**Migrant with Australian-born partner**	25 118 (29.4%)	1.03 (0.91–1.16)	1.01 (0.90–1.14)	0.99 (0.93–1.05)	0.97 (0.92–1.03)

*P<0.05

**Adjusted for maternal age group, maternal height, pre-existing medical conditions, parity, marital status, insurance status, socioeconomic disadvantage, smoking in pregnancy, year of birth and infant sex.

Note, each acculturative factor presented in this table was analysed separately.

Migrant women had 34% higher odds of term-LBW (aOR 1.34, 95% CI 1.24–1.44) than Australian-born women in the adjusted models. The odds of term-LBW in migrant women with an Australian-born partner was similar to that of the Australian-born women and the odds of term-LBW reduced in migrants with longer length of residence (Table 2). The odds of term LBW in migrant women with an Australian-born partner was similar to that of the Australian-born women (aOR 1.01, 95% CI 0.90–1.14). There was a gradual reduction in the adjusted odds of term LBW in migrant women as length of residence increased (<5 years, aOR 1.47, 95% CI 1.33–1.63; >10 years 1.22, 1.10–1.35).

**Fig 1:**
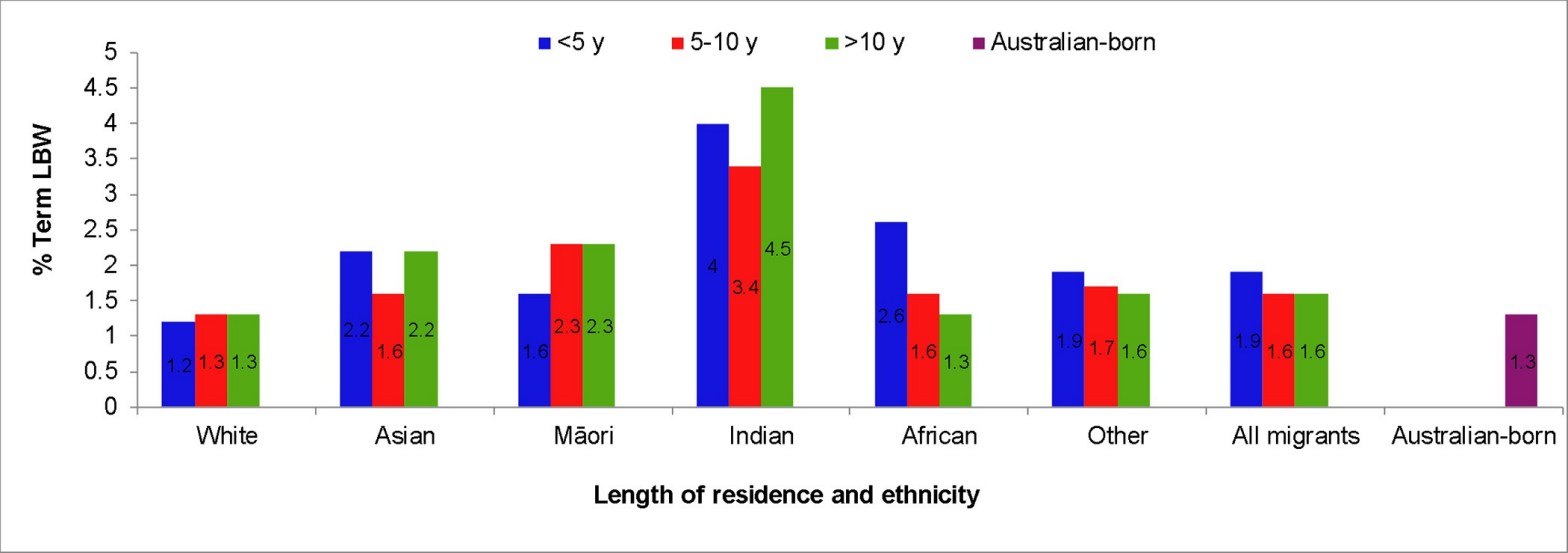
Term-LBW rate by ethnicity of migrants and the length of residence in Australia (2005–2013).

While the percentage of term-LBW decreased with longer length of residence in women from African and other ethnic backgrounds, it followed a U-shape pattern in births to Asian and Indian women and increased among Māori women residing in Australia for more than five years (Fig 1). Compared to Australian-born women, migrant women from Indian (OR 3.13, 95% CI 2.61–3.76), African (OR 2.07, 95% CI 1.55–2.76), Asian (OR 1.74, 95% CI 1.46–2.08) and ‘other’ (OR 1.49, 95% CI 1.17–1.91) backgrounds had significantly higher odds of term-LBW in the first five years of residence. Among those with 5–10 years residence, women from Indian and Māori backgrounds had higher odds of term-LBW (OR 2.64; 95% CI1.85–3.76, OR 1.78; 95% CI 1.03–3.09 respectively) than Australian-born women. For those with >10 years residence, women from Indian (OR 3.57; 95% CI 1.85–3.76), Māori (OR 1.79; 95% CI 1.15–2.80) and Asian (OR 1.70; 95% CI 1.42–2.04) backgrounds had higher odds of term-LBW outcome than their Australian-born counterparts. Adjusting for age and smoking significantly attenuated the odds of term-LBW in women from Māori background (aOR 1.20, 95% CI 0.76–1.87) but not in other groups.

**Fig 2:**
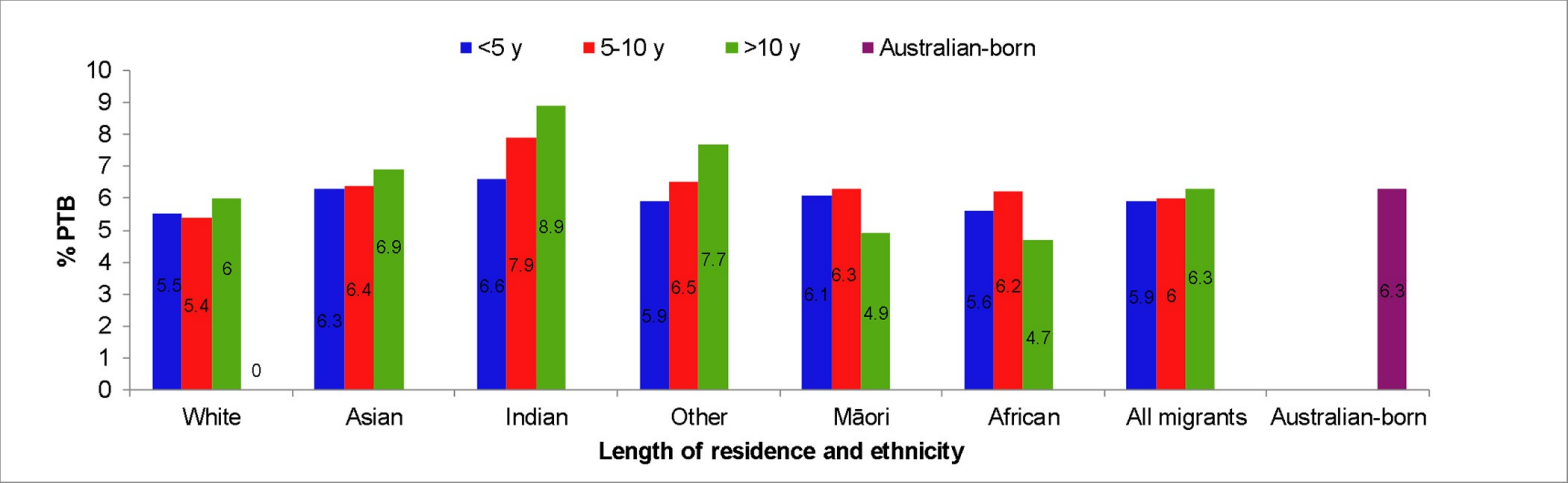
All PTB rate by ethnicity of migrants and the length of residence in Australia (2005–2013).

Proportions of PTB increased with longer length of residence in Australia for all migrants, except for those from Māori and African backgrounds (Fig 2). Among migrant women who had resided in Australia for more than ten years, the age and smoking adjusted odds of PTB in Māori women were 32% lower (aOR 0.68, 95% CI 0.50–0.92) while for women from Asian, other and Indian backgrounds the odds were 15%, 26% and 50% higher (aOR 1.15, 95% CI 1.04–1.2; aOR 1.26, 95% CI 1.083–1.46; aOR 1.50, 95% CI 1.137–1.98, respectively) than in Australian-born population.

### Level of acculturation

When acculturation was examined in multidimensional manners considering many proxies simultaneously and the spontaneous and medically-indicated PTBs were analysed separately, migrant women with an overseas-born partner who used a paid interpreter had significantly higher odds of term-LBW and lower odds of spontaneous and medically-indicated PTB than Australian-born women (Table 3). In contrast, for migrant women with an Australian-born partner, the odds of term-LBW, spontaneous PTB and medically-indicated PTB were similar to those of the Australian-born women (Table 3). For the least acculturated women, the odds of term-LBW were 58% higher than that of Australian-born women (aOR 1.58, 95% CI 1.15–2.18) while the odds of spontaneous PTB were 40% lower (aOR 0.60, 95% CI 0.45–0.80). In contrast, the most acculturated women had 43% (aOR 1.43, 95% CI 1.14–1.78) increased odds of spontaneous PTB and similar odds of medically-indicated PTB and term-LBW compared with their Australian-born counterparts (Table 3).

**Table 3:** Comparison of term-LBW, spontaneous and medically-indicated PTB in migrant and Australian-born women according to the acculturation level of migrant women.

Population	Term-LBW		PTB					
			All		Spontaneous		Medically-indicated	
	OR	aOR	OR	aOR	OR	aOR	OR	aOR
	(95% CI)	(95% CI)**	(95% CI)	(95% CI)**	(95% CI)	(95% CI)**	(95% CI)	(95% CI)**
**Australian-born**	1.00	1.00	1.00	1.00	1.00	1.00	1.00	1.00
**Migrant women with overseas-born partner and interpreter**	1.60*	1.65*	0.62*	0.54*	0.72*	0.66*	0.50*	0.44*
	(1.26–2.02)	(1.32–2.06)	(0.52–0.73)	(0.46–0.63)	(0.58–0.88)	(0.53–0.82)	(0.39–0.66)	(0.34–0.57)
**Migrant women with overseas-born partner but no interpreter**	1.49*	1.43*	0.98	0.95*	0.95	0.94*	1.02	0.96
	(1.38–1.61)	(1.32–1.54)	(0.94–1.02)	(0.92–0.99)	(0.90–1.01)	(0.88–0.99)	(0.96–1.07)	(0.91–1.02)
**Migrant women with Australian-born partner**	1.04	1.05	0.98	0.94*	0.99	0.99	0.97	0.94
	(0.92–1.17)	(0.94–1.17)	(0.93–1.04)	(0.89–0.99)	(0.92–1.07)	(0.92–1.07)	(0.90–1.05)	(0.87–1.02)
**Least acculturated** ***	1.54*	1.58*	0.56*	0.52*	0.64*	0.60*	0.47*	0.43*
	(1.13–2.11)	(1.15–2.18)	(0.44–0.70)	(0.41–0.65)	(0.48–0.86)	(0.45–0.80)	(0.33–0.67)	(0.30–0.61)
**Most acculturated** ****	1.16	1.11	1.37*	1.29*	1.48*	1.43*	1.24	1.15
	(0.77–1.74)	(0.74–1.67)	(1.15–1.62)	(1.09–1.53)	(1.19–1.85)	(1.14–1.78)	(0.97–1.60)	(0.89–1.48)

*P<0.05

**Adjusted for maternal age group, maternal height, marital status, pre-existing medical conditions, infertility treatment, insurance status, socioeconomic status, parity, smoking in pregnancy, year of birth and infant sex

***Least acculturated: non-white non-Māori women immigrated as an adult, had an overseas-born partner, lived in Australia <5 years, and used interpreter service; ****Most acculturated: non-white non-Māori women, immigrated as a child, lived in Australia >10 years, had an Australian-born partner and did not use interpreter service.

## Discussion

In this whole-population linked data study, we showed that disparities in the risk of PTB and term-LBW between migrant and Australian-born women differed according to the acculturative characteristics of the population. Migrant women who used the interpreter service, did not have an Australian-born partner, and had been in Australia <5 years (less acculturated) had lower odds of PTB than the Australian-born population. All migrants had a higher risk of term-LBW than the Australian-born women except for those with an Australian-born partner. Acculturation was associated with an increased the risk of spontaneous PTB but decreased the risk of term-LBW in non-white non-Māori migrant women.

### Low birthweight

We found that immigrants, regardless of their length of residence in Australia or whether they arrived as a child or adult, had a higher risk of term-LBW than the Australian-born group; however, the difference was higher in those who had been in Australia for <5 years and/or immigrated as adults. This finding is similar to previous observations in Australia, but findings from other nations are equivocal [3, 34]. Hyman and Dussault in 1996 reported increased odds of term-LBW for Asian women and decreased odds for Italian and Greek migrant women living in Canada, implying that higher odds were associated with a higher level of acculturation [34, 35]. Martinson, Tienda and Teitler in 2017 reported that foreign-born status (collectively) protected against LBW among migrants residing for ≤5 years in the US and the UK but observed an increased odds of LBW (OR = 1.29), although non-significant, among migrants residing in Australia for ≤5 years [3]. They reported that the three countries studied had a similar overall pattern of LBW by duration of residence and in their final multivariable analysis, the risk was highest during the first two years following resettlement. However, the results differed for migrants from different ethnic backgrounds; African and Asian migrant women resettled in the UK had 3.9–4.3 times higher odds of having a LBW baby than immigrant mothers from English-speaking countries, while in Australia, women from Asian and other low-income countries had 4.5–7.2 times higher odds of LBW babies than their counterparts from Anglophone nations. It is worth noting that this study was based on a sample of 3732 participants from the Australia, and lacked information on self-reported ethnicity; unlike our study population (*N* = 252 256) with 100% completeness for ethnicity. Thus, small sample size may have reduced the statistical power to detect this effect in their study. The higher risk of LBW in the first few years after immigration may be a result of stressors related to immigration, acculturative and financial instabilities experienced by migrants [36].

With a longer length of residence, the rate of term-LBW decreased among women from African and other ethnic backgrounds in our study, increased among Māori women residing in Australia for more than five years, and followed a U-shape pattern in births to Asian and Indian women. Similarly, a U-shaped pattern for associations of LBW with the length of residence for immigrant groups was seen in Denmark [37] and with time since naturalisation for immigrants residing in Belgium [38]. Teitler, Hutto and Reichman have also reported a curvilinear association pattern in infants’ birthweight by maternal duration of residence in the US for both immigrants overall, and for Hispanic immigrants in particular [39]. This finding suggests that the ‘healthy migrant paradox’, which implies better initial health outcomes for migrants than the host population that then deteriorate with time [17, 40] may not be applicable for LBW risk to all migrant groups, and the change in the risk of LBW over time may not be linear in either direction. The initial decrease observed in the risk may be explained by an improvement in employment and financial status in the short term. We previously reported a considerable rise in the proportion of those with private health insurance in migrant women from white, Asian and Indian ethnic backgrounds in Australia [22]. On the other hand, the decline over time may be due to the accumulation of stressors related to discrimination, acculturation and unhealthy lifestyle choices, such as smoking, among migrants [22, 38].

We also showed that having an Australian-born partner was significantly associated with a lower risk of LBW in migrants. A lower risk of stillbirth for migrant women with a non-migrant partner than those with overseas-born partners has been reported in Australia, Norway and the US, as well as a lower risk of small-for-gestational-age in Chinese-American women with white or Black partners in the US, compared to those with Chinese-American partners [22, 41–43]. Paternal genetic impact [44, 45], fathers’ lifelong socioeconomic position [46] and/or factors determining maternal environmental and lifestyle circumstances, including stress or social support and dietary or smoking habits [18, 35], influenced by acculturation, all may play a role. Given that intermarriage is a strong predictor of integration in a multicultural society [47], migrant women with an Australian-born partner may experience an enhanced social support and a favourable environment compared to their migrant counterparts who have an overseas-born partner. Previous studies have highlighted the impact of acculturative stress, lack of support and the increased likelihood of experiencing marriage conflict and even domestic violence for migrant women with migrant partners [48–50].

### Preterm birth

Migrant women who immigrated as a child, women who had a >10 year length of residence, and women with an Australian-born partner were similar to the Australian-born population in the risk of PTB. When the migrant population was stratified by ethnicity, among Asian, Indian and ‘other’ migrants, the risk of PTB was significantly higher in those with longer length of residence. Further, when level of acculturation, among the non-white non-Māori migrant women collectively, was determined by considering all proxies simultaneously, acculturation was associated with a higher risk of PTB. Similarly, in Canada, recent immigrants were at lower risk of PTB, but the risk was higher in those with >10 years of residence than their Canadian-born counterparts [51]. A higher level of acculturation was significantly associated with higher odds of preterm birth in the US [20, 52]. The influence of acculturation on the risk of PTB among migrant groups may be explained by the stressors experienced and/or the change of health behaviours in migrant populations. The evidence suggests that women with higher levels of cortisol are at higher risk of experiencing PTB [53]. Cortisol secretion is influenced by stress, and researchers have investigated the relationship between acculturative stress and cortisol levels in migrants [54, 55]. Ruiz et al. showed that the more acculturated women were more likely than the less acculturated women to exhibit higher stress responses measured by cortisol in blood samples [54]. Nicholson et al. showed, through diurnal cortisol response measured by area-under-the-curve analysis and using salivary samples, a higher level of proficiency in English language in former Soviet immigrants residing in the US produced an increased cortisol response [55]. Higher levels of acculturation were also associated with attenuation of the cortisol awakening response in Mexican-American adults [56]. A flattened or blunted cortisol awakening response has also been reported in Indigenous participants and is believed to be associated with chronic experience of stress [57]. Furthermore, progesterone is an important hormone for maintaining pregnancy and decreased levels have been linked to preterm birth [58]. On the other hand, greater English proficiency in Hispanic women, hence higher acculturation level, has been linked to a decrease in progesterone levels and predicted lower gestational age [20]. Thus, we believe that hormonal alterations in response to acculturative stress may explain the findings we observed in our study.

### Strengths and limitations

Our study is the first comprehensive investigation to explore the influence of acculturation on the risk of PTB and term-LBW in migrants from diverse ethnic backgrounds in Australia. The large size of the study—including all migrants and non-Indigenous Australian-born women giving birth—having access to numerous variables from linked databases and registries, and the methodology of a whole-population retrospective cohort design, which eliminates the risk of selection, participation and recall biases, strengthened the robustness of the study and the reliability of our findings. The statutory and self-reported nature of our data, mandating registration of child’s birth within 60 days of the birth by submitting a Birth Registration Form completed and signed by both parents, resulted in high accuracy of data. Using term-LBW as the outcome of interest, instead of the crude measure of LBW, eliminated the possible bias due to the influence of prematurity on birthweight of those born preterm.

We acknowledge, however, that our study has some limitations. We used routinely collected administrative health data that had not been collected specifically to answer the research questions of this specific study. Nevertheless, the WADLS was originally established as a result of collaboration between the WA Department of Health and researchers, mainly for population health research [24]. The WADLS has also been extensively validated and the quality of the linkages is high with very low error rates [26, 27]. The MNS data and the variables we used have also been previously validated [59]. However, we recognise that such data collections can be improved to include more information on paternal and other characteristics as we were not able to separate the partners’ ethnicity or to investigate whether they were second generation migrant given the current data. Also, we separated spontaneous PTB from those medically indicated, however, it should be noted those individuals with preterm pre-labour rupture of membranes who gave birth before completed 37 weeks of gestation have not been separated under a distinctive PPROM category in this paper.

Further, acculturation is a dynamic process, and its influence on health behaviour and outcomes is multifactorial. While we, similar to other population studies, examined acculturation effect using proxies and not through interview or survey, efforts were made to undertake the investigation in a multidimensional manner using multiple variables, self-reported ethnicity, language proficiency, age on arrival, intermarriage and length of residence. This approach helped to provide useful information for a large and diverse population of migrants and can be used to inform policy and practice and further investigation.

In conclusion, our data provided evidence that recent migrants had a higher risk of term-LBW and lower risk of spontaneous and medically-indicated PTB than the Australian-born population in their first few years after immigration. This may indicate that in this population the growth restricted babies were not well identified in pregnancy or did not receive appropriate interventions as their Australian-born counterparts. We also showed that acculturation had positive effects on the risk of term-LBW but was associated with a higher risk of spontaneous PTB in migrant women in Australia which may be due to acculturative stresses experienced by migrants. This pattern, however, may differ among specific ethnic groups. Nevertheless, our findings suggest that acculturation level and related characteristics are important factors that should be considered in pregnancy care of migrant women. This emphasises the need for understanding the process of acculturation and its health impact to plan the interventions required for prevention of PTB and LBW in migrants and ethnic minorities at risk.

Our findings can be generalised to other populations hosting migrants from similar ethnic backgrounds; however, care should be taken with interpretation of the results due to factors such as different immigration and citizenship laws, healthcare systems, cultures and lifestyle norms in host countries.

## Supporting information

S1 Checklist STROBE statement—checklist of items that should be included in reports of *cohort studies*. (DOCX)

S1 File Choosing confounding variables using DAGs. (PDF)
