# Supplementary material for: The influence of acculturation on the risk of preterm birth and low birthweight in migrant women residing in Western Australia
Source: PLoS One. 2023 May 10;18(5):e0285568. doi: 10.1371/journal.pone.0285568 (PMC10171663; doi:10.1371/journal.pone.0285568)
Supplement: S1 File — (PDF) [file pone.0285568.s002.pdf]

The influence of acculturation on the risk of preterm birth and low birthweight in migrant women residing in Western Australia

Supplementary Document

Statistical analysis

Choosing the confounding variables

The process of integration and acculturation is complex and many subtle and evident processes are in play at once. The population of migrants are heterogeneous with some having better socioeconomic status and some worse, depending on their immigration route, being a skilled worker, a student or a refugee, compared to their counterparts in the host country.

As such, it is difficult to draw the associations using Directed Acyclic Graphs (DAGs) for such a complex chain of associations. However, to accommodate a reviewer’s comment we attempted to also use the DAGs method for selecting the confounders, using DAGitty.net, as is shown below. We then identified the minimal sufficient adjustment sets and removed from the multivariable logistic regression the extra variables. These included infant sex and maternal height.

Removing these variables did not change the result statistically and adjusting for them did not introduce any bias according to the DAG model. Hence, we kept all the variables in the analysis.

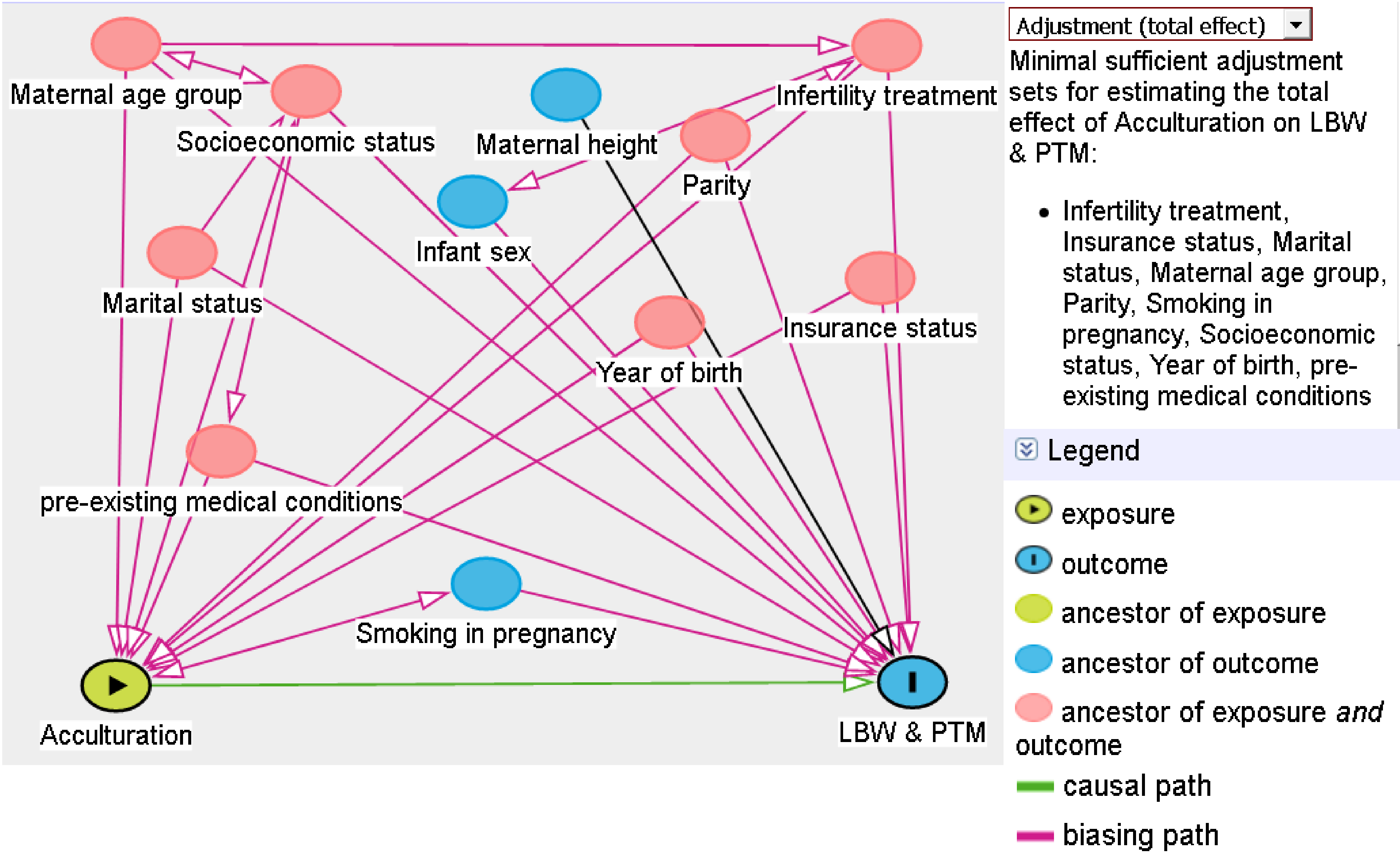

Directed acyclic graph (DAG) for the association between Acculturation and LBW & PTB

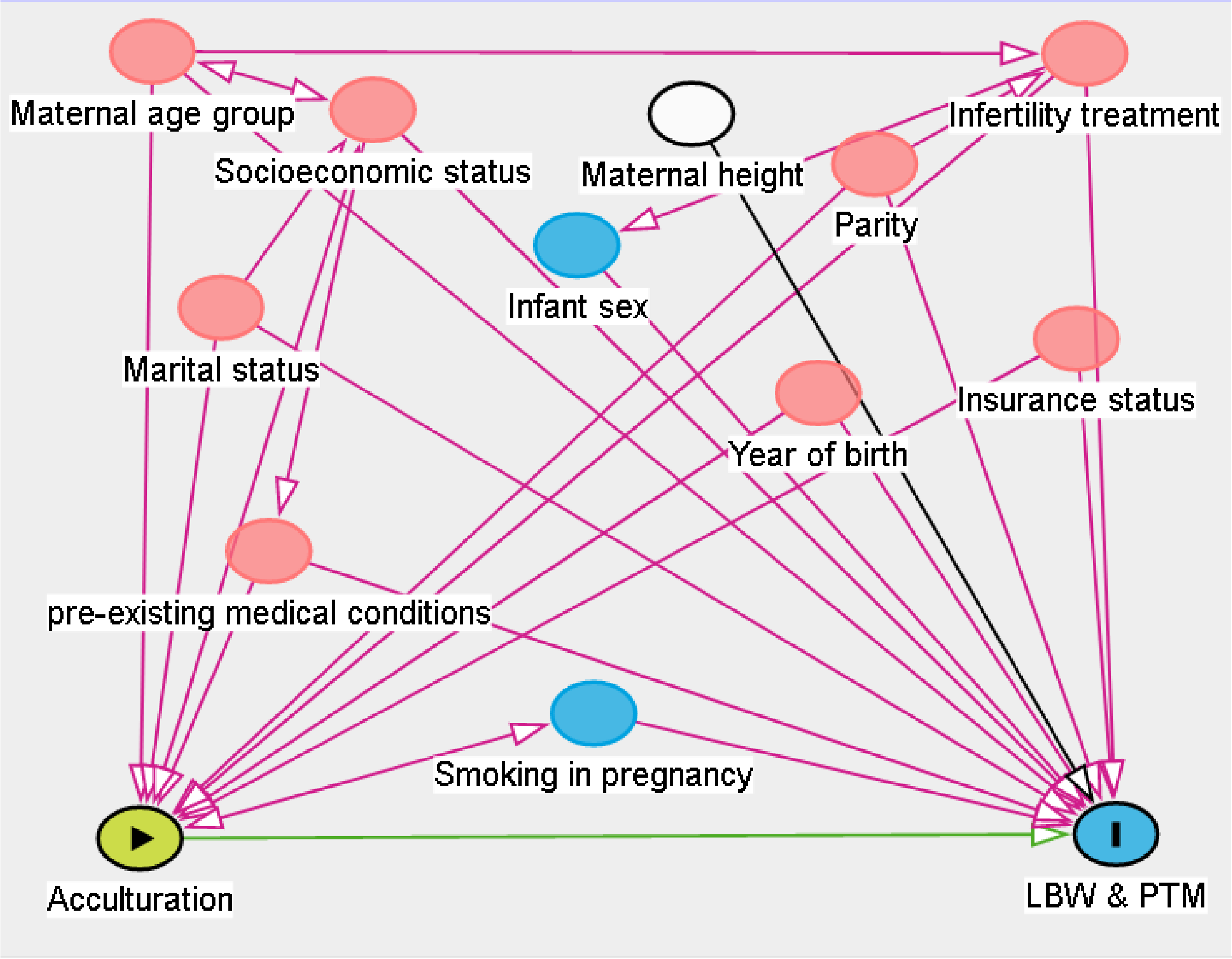

Causal effect identification

Adjustment (total effect)

Minimal sufficient adjustment sets containing Maternal height for estimating the total effect of Acculturation on LBW & PTM:

- Infertility treatment, Insurance status, Marital status, Maternal age group, **Maternal height**, Parity, Smoking in pregnancy, Socioeconomic status, Year of birth, pre-existing medical conditions

Legend

- exposure
- outcome
- ancestor of exposure
- ancestor of outcome
- ancestor of exposure *and* outcome
- adjusted variable
- causal path
- biasing path

Maternal height as a confounder was suggested by a reviewer and In the regression analysis was strongly associated with PTB & LBW

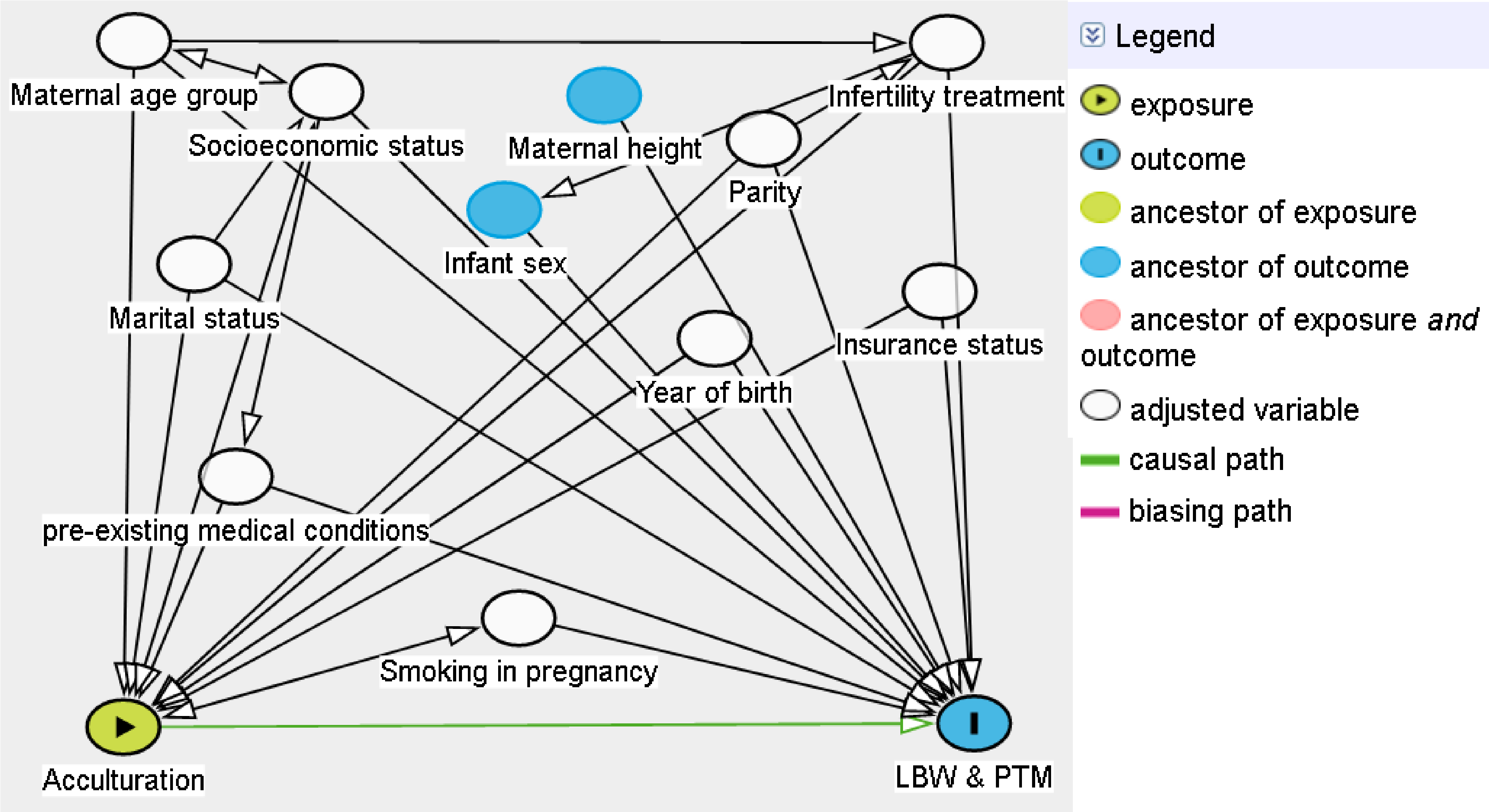

After adjusting for the minimal sufficient adjustment sets

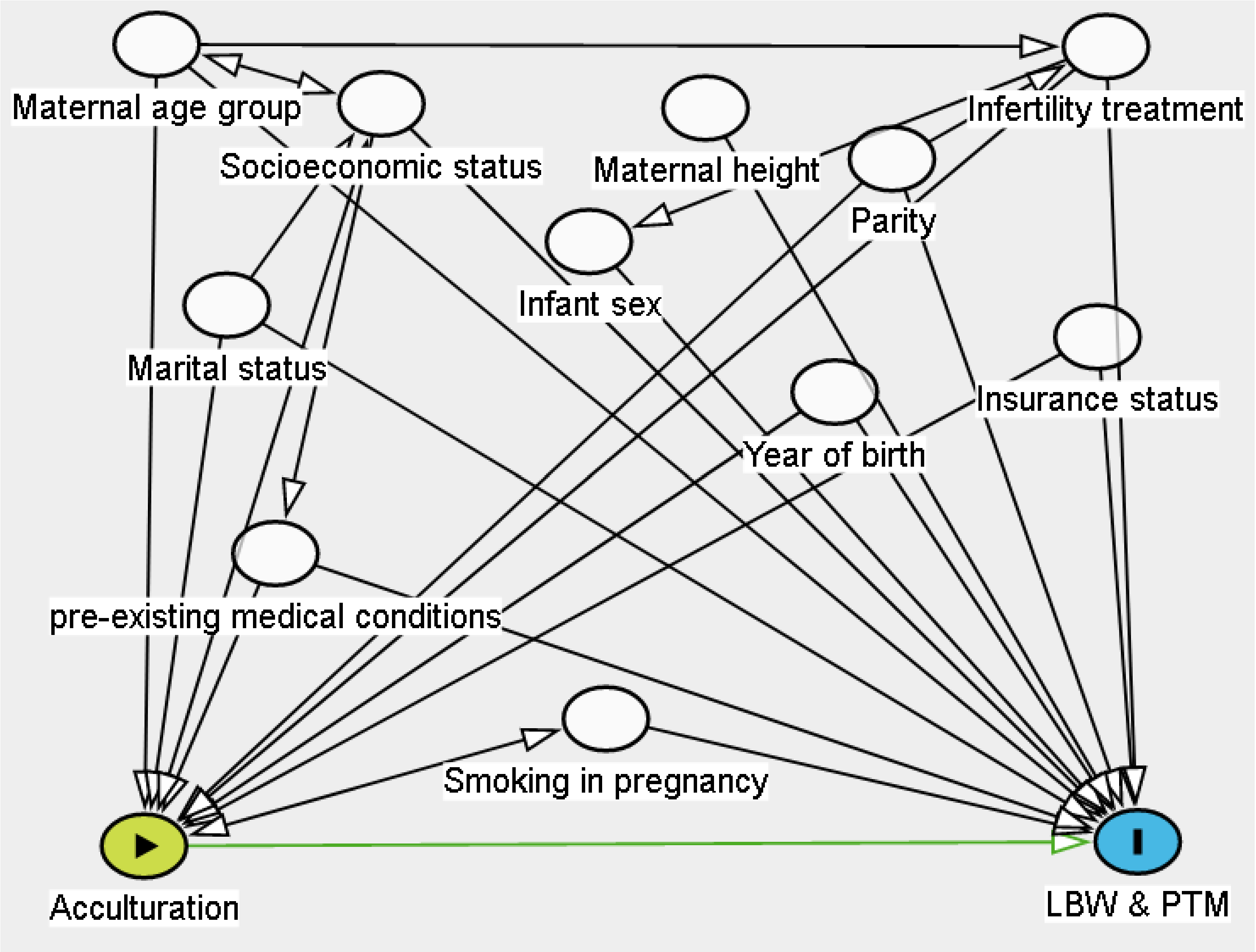

After adjusting for maternal height and infant sex in addition to the minimal sufficient adjustment sets: No bias was introduced
